# CMTF: Control-Point Guided Motion Transformer Framework for Robust Trajectory Prediction

**DOI:** 10.3390/s26206412

**Published:** 2026-10-10

**Authors:** Zishan Zhao, Haifeng Sang

**Affiliations:** School of Information Science and Engineering, Shenyang University of Technology, Shenyang Economic and Technological Development Zone, No. 111 Shenliao West Road, Shenyang 110870, China; sanghaif@163.com

**Keywords:** trajectory prediction, Out-of-Distribution generalization, Bernstein polynomial, geometric consistency loss

## Abstract

Trajectory prediction in autonomous driving must achieve both high accuracy and strong generalization to cope with complex and dynamic real-world traffic environments. However, existing methods are trained predominantly on in-distribution data from a single dataset, and their performance degrades severely in Out-of-Distribution (OoD) scenarios. To address this limitation, this paper proposes the Control-Point Guided Motion Transformer Framework, CMTF, a general framework for trajectory prediction that improves robustness and interpretability under OoD conditions. CMTF builds on the classical Bernstein polynomial parameterization and represents each trajectory as a small set of control points that carry explicit spatial semantics. The key novelty of this work lies in a shift of the supervision paradigm. Control points are elevated from passively used auxiliary encoding features in prior methods to explicit global supervision signals. These signals operate throughout the entire framework, from structured input augmentation to the auxiliary prediction head and the geometric consistency loss, and guide the model to learn trajectory representations with clear structure and geometric plausibility. Without increasing the amount of training data, CMTF can be integrated into mainstream trajectory prediction models. Cross-dataset evaluations on nuScenes, Argoverse2, and WOMD show a consistent trend of improved OoD performance.

## 1. Introduction

To ensure the safety and reliability of autonomous driving systems, autonomous vehicles must accurately perceive and understand the interactions among the ego vehicle, the surrounding environment, and other traffic participants [1]. Central to this perception is the prediction of the trajectories of surrounding traffic participants, which enables the system to anticipate potential risks and to make informed decisions in navigation, speed adjustment, and obstacle avoidance. The trajectory prediction task infers future motion patterns from historical trajectory data, environmental context, and real-time observations. The development and evaluation of current state-of-the-art trajectory prediction models [2,3,4] have focused primarily on In-Distribution (ID) performance within a single dataset. Here, ID evaluation refers to training a model on the training split of a dataset $D_{s}$ and evaluating it on the test split of the same dataset. Out-of-Distribution (OoD) evaluation refers to cross-dataset transfer, in which the model is trained only on the source domain $D_{s}$ and tested on a target domain $D_{t}$ from a different source, with $D_{s}\neq D_{t}$ and without fine-tuning on target-domain samples. However, test samples and training samples within a dataset still share considerable similarities in aspects such as sensor configurations, map representations, post-processing pipelines, geographic distributions, and scenario selection biases. The strong performance of a model in this setting therefore reflects its adaptation to the characteristics of a single source domain rather than genuine generalization, and may indicate a form of overfitting to a particular distribution. In practical applications, the performance of trajectory prediction should be assessed through cross-dataset OoD testing rather than through reliance on the shared biases of a single dataset. In this study, we therefore unify data formats and prediction tasks to enable OoD evaluation on large-scale motion datasets, namely nuScenes [5], Argoverse2 [6], and WOMD [7]. Building on these OoD evaluations, we further explore the possibility of enhancing the generalization of models to OoD samples.

One strategy that can enhance the generalization capability of any deep learning model is to increase the quantity of training data [8]. However, manual annotation of ground truth is often costly and time-consuming and is prone to inconsistency and subjectivity. One remedy is to algorithmically generate new data to expand the dataset [8,9,10]. Generating more data, however, requires additional storage resources and training time. This study therefore proposes an alternative strategy that introduces an auxiliary supervision mechanism based on the geometric structure of trajectories without increasing the volume of the original data. Specifically, we employ Bernstein polynomials to parameterize trajectories. The coefficients of these polynomials naturally carry spatial semantics and can be interpreted as control points that govern the trajectory shape. In contrast to discrete trajectory points, these control points are not tied to specific time steps. They modulate the geometric form of the trajectory in a global manner and provide clear geometric interpretability. Control points can thus be viewed as latent turning nodes that reflect the future behavioral intentions of a vehicle. Building on this property, we construct structured control-point supervision signals that guide the model to focus on key geometric features of decision-making significance, thereby enhancing its ability to model and generalize future trajectory trends. These control points not only improve the geometric consistency of trajectory representations but also provide semantically meaningful decision anchors for subsequent planning and control modules, which helps the system react more reasonably in dynamic environments. Throughout this paper, geometric consistency refers to the degree of shape agreement between the predicted trajectory and the reference trajectory obtained through Bézier interpolation of the ground-truth control points. This property derives from the inherent smoothness and convex-hull property of the Bernstein basis functions and from the structural alignment introduced through differentiable reconstruction. The corresponding constraint acts on the smoothness and geometric plausibility of the trajectory.

Conventional data preprocessing first extracts the positions, dimensions, heading angles, and velocity validity flags of trajectories and processes time windows through fixed step sizes and sampling intervals, after which input features comprising historical trajectories, future trajectories, and map features are constructed. However, this representation paradigm suffers from limitations that constrain model performance and practical applicability. Specifically, the acquisition process of discrete sequence-based representations, which involves collection, sampling, and interpolation, introduces discontinuities. As a consequence, the measurement noise and outliers inevitably present in trajectory data tend to carry high-frequency information [11], which substantially increases the risk of model overfitting. In essence, such models merely memorize patterns in the data rather than truly understand the underlying motion regularities, which limits their applicability in complex real-world scenarios. To address these challenges, this study proposes to explicitly model the key geometric features of trajectories, strengthening the ability of the model to understand trajectory shapes and to capture complex trajectory forms more accurately. In addition, the model is required to predict the timing of the critical control points at which the decisions of the target vehicle are likely to change, which enables the proactive perception of key geometric features in future trajectories.

To address the insufficient generalization of existing trajectory prediction models in real-world scenarios and the high cost of data generation, this paper proposes a control-point guided general framework for trajectory prediction. To clearly delimit the contribution of this framework relative to existing trajectory representation methods, we clarify that Bernstein basis functions and their least-squares fitting are classical tools in computer-aided geometric design. The core innovation of the framework instead lies in a shift of the supervision paradigm, which elevates control points from auxiliary encodings in prior methods to active and explicit global supervision signals. The main contributions are as follows:

1. Full-trajectory control-point parameterization and explicit global supervision: Unlike prior methods that impose control-point supervision only at selected time steps or employ control points as unconstrained auxiliary encodings or intermediate features, this work parameterizes the entire future trajectory into a continuous control-point sequence through Bernstein polynomials. The resulting sequence serves as a global supervision signal that carries spatial semantics, remains differentiable, and acts as a regression target, thereby improving the modeling of trajectory shapes from the perspective of geometric structure.
2. Pluggable closed-loop control-point supervision mechanism: We propose a closed-loop supervision mechanism composed of three components: structured pseudo-trajectory input augmentation, an auxiliary control-point prediction head, and a geometric consistency loss based on differentiable Bézier reconstruction. This mechanism explicitly decouples trajectory shape from sampling noise, in which control points carry the global geometric intent while trajectory points ensure execution accuracy. The mechanism is decoupled from the backbone and can be integrated into mainstream prediction architectures such as MTR [12] and AutoBot [13] without modifying the backbone structure.
3. Systematic validation of OoD generalization without data augmentation: We unify the testing protocol across three large-scale public datasets, namely nuScenes, Argoverse2, and WOMD, and conduct both ID and OoD evaluations. These experiments systematically verify the consistent effectiveness of substituting structural supervision for data expansion in complex scenarios and under cross-domain conditions.

## 2. Related Work

Driven by substantial progress in sensing technology, artificial intelligence, and automatic control systems, autonomous driving has become an effective solution for reducing traffic accidents and alleviating traffic congestion [14]. Although autonomous vehicles have achieved remarkable advances in perception, decision-making, planning, and control systems, safety concerns still restrict their application and popularization. One key technology for improving driving safety is trajectory prediction, which endows autonomous vehicles with the ability to forecast the future states of traffic participants in real time, to identify and assess potential hazards in advance, and to improve the effectiveness of decision-making and planning [15]. Nevertheless, safety and generalization remain the main bottlenecks that limit system deployment.

In recent years, researchers have systematically reviewed discrete sequence-based trajectory prediction for autonomous driving from complementary perspectives. Madjid et al. [16] surveyed the progress, limitations, and future directions of trajectory prediction for autonomous driving and identified trajectory representations that generalize across datasets as a pressing open problem. Yin et al. [17] reviewed deep-learning-based vehicle trajectory prediction and emphasized that incorporating vehicle motion characteristics beyond purely data-driven fitting markedly improves reliability and interpretability. This view aligns with the spatial geometric semantics of the control-point representation adopted in this work. Bosurgi et al. [18] analyzed the performance indexes used to characterize driving behavior and identified the diversity of driving styles as a major source of prediction difficulty. The structured control points in this work address this challenge by capturing the global geometric intent of a trajectory rather than local sampling noise. Yan et al. [19] surveyed typical applications of vehicle trajectory prediction in autonomous driving and noted that cross-domain generalization remains an open challenge that constrains practical deployment. Li et al. [20] proposed an end-to-end trajectory prediction method for unmanned ground vehicles based on multimodal fusion, which combines a BEVFormer perception model with knowledge distillation to achieve high-precision and low-latency prediction. Zhang et al. [21] introduced MTP-STG, a spatio-temporal graph Transformer network for multimodal trajectory prediction in crowd scenarios that models scaled spatial interactions through graph attention. Suk and Kim [22] developed an uncertainty-aware multimodal trajectory prediction model that quantifies both aleatoric and epistemic uncertainties in a single forward pass, which underscores the demand for robust trajectory representations under uncertainty. By replacing high-frequency discrete trajectory points with smooth Bernstein polynomial control points, this work inherently suppresses observation noise at the representation level and offers a new perspective for such demands.

At present, the development and evaluation of state-of-the-art trajectory prediction models [23,24,25] concentrate mainly on ID performance within a single dataset. These methods perform well under standard evaluation protocols but tend to overfit the scenario biases of a particular dataset, such as map layouts and sensor configurations, and their generalization to OoD samples lacks systematic verification. OoD testing examines the ability of a model to perform well on data whose distribution differs from that of the training data. However, OoD testing faces challenges arising from inconsistent data formats and task details, such as differences in scenario length, observation history, and prediction horizon. Feng et al. [2] unified the formats of multiple datasets to lay a foundation for cross-dataset evaluation and released the largest public dataset for training trajectory prediction models, concluding that larger and more diverse datasets markedly improve model performance and generalization. Larger datasets, however, entail considerable storage and training costs. A unified mechanism for improving robustness against the model degradation that occurs under OoD shifts is still lacking.

In addition, data-driven methods can extract vehicle behavior information from complex traffic environments, but do so at the cost of interpretability. Because of their black-box nature, data-driven approaches are frequently questioned regarding the interpretability and reliability of their trajectory predictions. To meet this challenge, several studies introduce domain knowledge as priors into model architectures or loss functions. Such priors provide additional constraints when data are limited, reduce the likelihood of implausible predictions, and enhance model interpretability. However, these knowledge-injection methods typically depend on specific rules or dynamics models and still face challenges in generality and scalability.

In recent years, Physics-Informed Deep Learning (PIDL) has emerged as a hybrid machine learning paradigm and has been widely applied in various domains [26,27,28,29]. By incorporating physical knowledge or domain priors into predictive models, PIDL yields hybrid models that combine interpretability with accuracy [30]. Recent studies show that embedding vehicle dynamics models into deep neural networks ensures that generated trajectories are physically feasible [31,32,33]. Bahari et al. [34] proposed a knowledge-driven trajectory prediction framework that uses residual learning to complement data-driven methods and applies model predictive control to constrain outputs in accordance with kinematic requirements. Nevertheless, these methods concentrate mostly on rule-based modeling. They lack the ability to model the global structure of trajectory shapes and therefore struggle to capture the critical turning information that arises during prediction.

At the representation level, polynomial trajectory modeling has attracted growing attention. Previous studies have shown that low-degree polynomials derived from curve parameterization fit real-world trajectories effectively and offer favorable smoothness and differentiability [35,36]. Moreover, their parameters naturally carry spatial semantics and can be interpreted as control points that govern the trajectory shape [8,11]. Nevertheless, most methods still treat control points as auxiliary encodings or intermediate features [37] and lack systematic supervision mechanisms and structural consistency modeling. Control-point prediction therefore remains unconstrained and can hardly guide trajectory generation in a stable manner. Existing methods also face a modeling trade-off. Direct trajectory-point prediction achieves high accuracy but weak structure, whereas control-point modeling provides strong structure but suffers from error propagation. An ideal strategy combines the two, with control points supplying high-level planning intent and trajectory points ensuring execution accuracy. Proactively capturing the latent turning points at which the decisions of the target vehicle change enables more accurate prediction and planning. In this work, control points are instead elevated into active supervision signals that operate throughout the training pipeline. Structured pseudo-trajectories augment the observations at the input side, an auxiliary prediction head explicitly regresses the control points at the output side, and differentiable Bézier reconstruction imposes a geometric consistency constraint at the loss side. Together, these components form a closed loop of structural supervision.

## 3. Methodology

### 3.1. Problem Definition

This paper proposes a novel approach for vehicle trajectory prediction by introducing trajectory control-point supervision and parameterized trajectory generation. Specifically, we consider an observed sequence of states from historical time steps t−h to *t*, denoted as $X_{\mathrm{tar}}=\left\{s_{t-h},\ldots ,s_{t}\right\}$, where *s* represents lightweight information acquired via onboard sensors, including position, velocity, vehicle heading, and dimensions. Moreover, map information *M* is utilized to impose constraints on the motion of the Target Vehicle (TV). The objective is to predict the future state sequence from time step t+1 to t+f, denoted as $Y_{\mathrm{tar}}=\left\{s_{t+1},\ldots ,s_{t+f}\right\}$.

Unlike conventional methods that directly predict the position at each future time step, this work introduces a parameterized representation of the trajectory, in which the target trajectory is modeled as a smooth curve defined by a limited number of control points. Specifically, a set of control points $C_{t,t-h}=\left\{c_{0},\ldots ,c_{n}\right\}$ is introduced to govern the geometric shape of the entire trajectory. The future trajectory points $Y_{\mathrm{tar}}$ are then modeled as a function supervised by the control points, formally expressed as follows:

(1) $$Y_{\mathrm{tar}}=D\left(X_{\mathrm{tar}},C_{t,t-h},M\right)$$

where D(·) denotes the trajectory prediction procedure that maps the historical trajectory and the control point set to future trajectory predictions.

According to the Akaike Information Criterion (AIC) [38], as adopted in [35], the historical trajectories of vehicles in the dataset can be optimally represented by a quintic polynomial. Specifically, the control point set over the temporal window [t−h,t] is defined as $C_{t,t-h}=B\left(p_{t-h},\ldots ,p_{t}\right)$, where B(·) denotes the Bernstein basis mapping that transforms the discrete position sequence into a continuous parametric representation. In practice, the raw position sequence $\left(p_{t-h},\ldots ,p_{t}\right)$ is first smoothed by a standard Kalman filter before the Bernstein mapping, following the preprocessing practice adopted in [36]. Operating as a linear state estimator on the noisy measurements, the Kalman filter suppresses high-frequency observation noise and yields consistent smoothed state estimates, thereby making the subsequent control-point fitting robust to measurement noise. The detailed derivation of this formulation is provided in Section 4.2.2. Here, $C_{t,t-h}$ should be interpreted as the control point set corresponding to the sub-trajectory from time t−h to *t*, which characterizes the local geometric structure of the motion.

During training, the control points serve not only as part of the implicit trajectory generation process but also as explicit auxiliary supervision signals, thereby enhancing the ability of the model to capture the structural characteristics of trajectories. The objective is to train the target predictor on the training set $D_{\mathrm{train}}$ so that it also achieves favorable performance on test set samples $D_{\mathrm{test}}$ beyond the source domain.

### 3.2. Control-Point Guided Motion Transformer Framework

**Figure 1:**
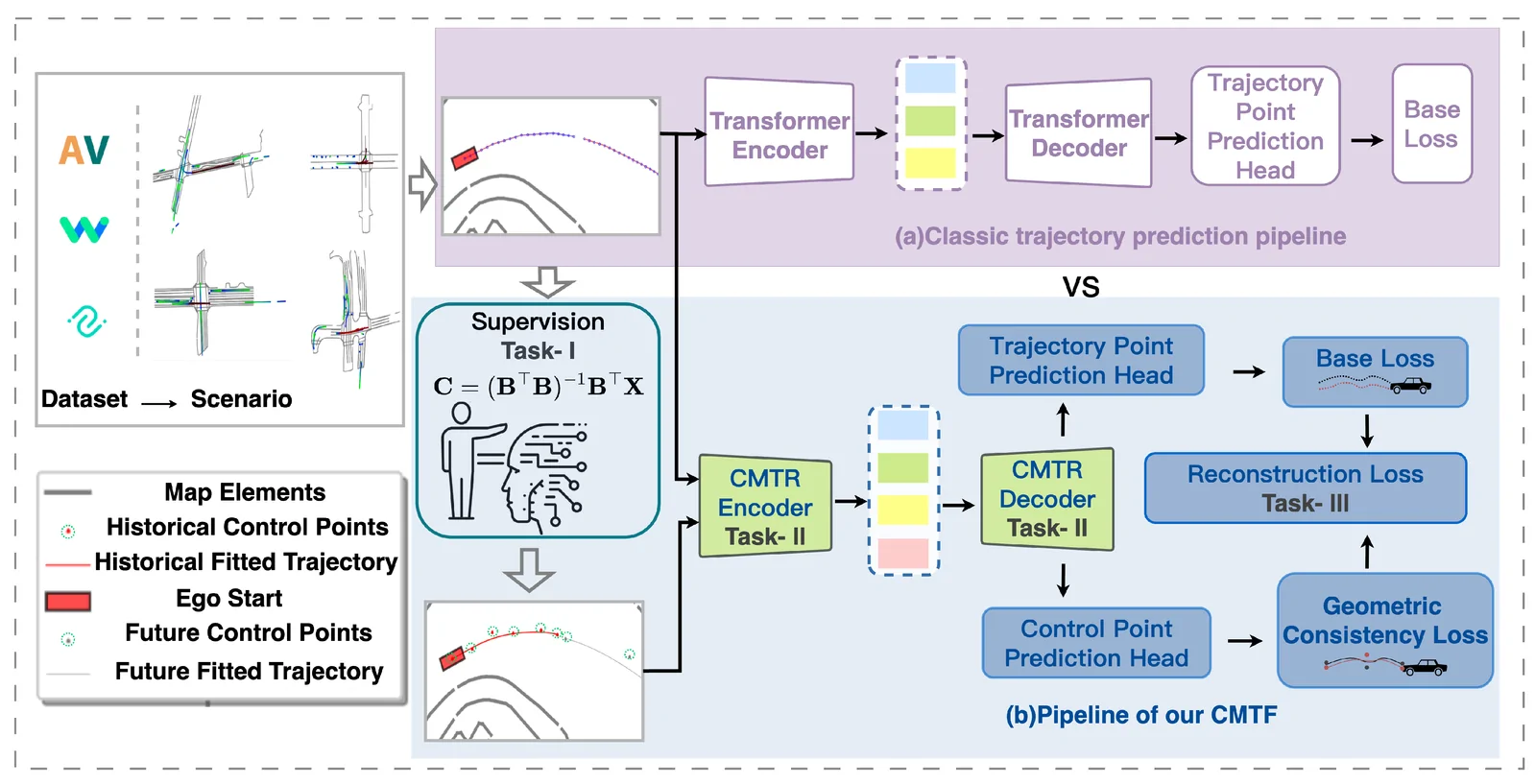
Comparison of (**a**) the traditional trajectory prediction pipeline and (**b**) the proposed CMTF.

The Control-Point Guided Motion Transformer Framework (CMTF) is a plug-and-play trajectory prediction enhancement framework designed for seamless integration into existing prediction models. As illustrated in Figure 1, compared with the conventional trajectory prediction pipeline (a), CMTF (b) introduces a control-point representation together with a novel supervision mechanism. CMTF comprises three task-specific modules, which correspond to control point generation and supervision signal construction, control-point-aware backbone modeling fusion, and control-point-guided prediction consistency optimization. These modules operate collaboratively through an explicit control-point representation and form an end-to-end structurally constrained learning pipeline. The framework is intended to endow the prediction model with stronger OoD generalization capability and geometrically smooth and structurally reasonable trajectory prediction:

1. Control point generation and supervision signal construction: The historical control points of the target vehicle are extracted from the observed historical trajectories to form structured geometric inputs that are available in both the training and inference stages. In addition, the ground-truth future control points are fitted from the future ground-truth trajectory only during training and serve as supervision signals for the auxiliary prediction head and the geometric consistency loss (I III).
2. Control-point-aware backbone modeling fusion: The interpolated trajectory generated from the historical control points serves as a pseudo-trajectory input, which is subsequently fused with the historical states and map features. This fusion strengthens the ability of the model to capture the global structure and geometric consistency of trajectories. An auxiliary mechanism is further devised to supervise the control information, guiding the main model to focus on key structural regions during the encoding stage (I II III).
3. Guided prediction and consistency supervision: The model explicitly regresses the future control points through an auxiliary prediction head. During training, the trajectory generated from the predicted control points is compared with the reference trajectory interpolated from the ground-truth future control points to construct a structural consistency loss. During inference, the historical control points are used to construct the input, and the prediction head outputs the future trajectory together with its probability (III II).

#### 3.2.1. Data Structure

The CMTF relies on an extended dataset that comprises both original observed trajectory pairs and fitted control-point annotation pairs. This dataset not only retains the conventional feature vector–label pairs used for trajectory prediction in data-driven methods but additionally constructs data pairs composed of control-point coordinates following the defined extraction and computation procedures. For ease of reference, Table 1 lists recurring variables in advance, and each symbol maintains a consistent meaning throughout the paper.

**Figure 2:**
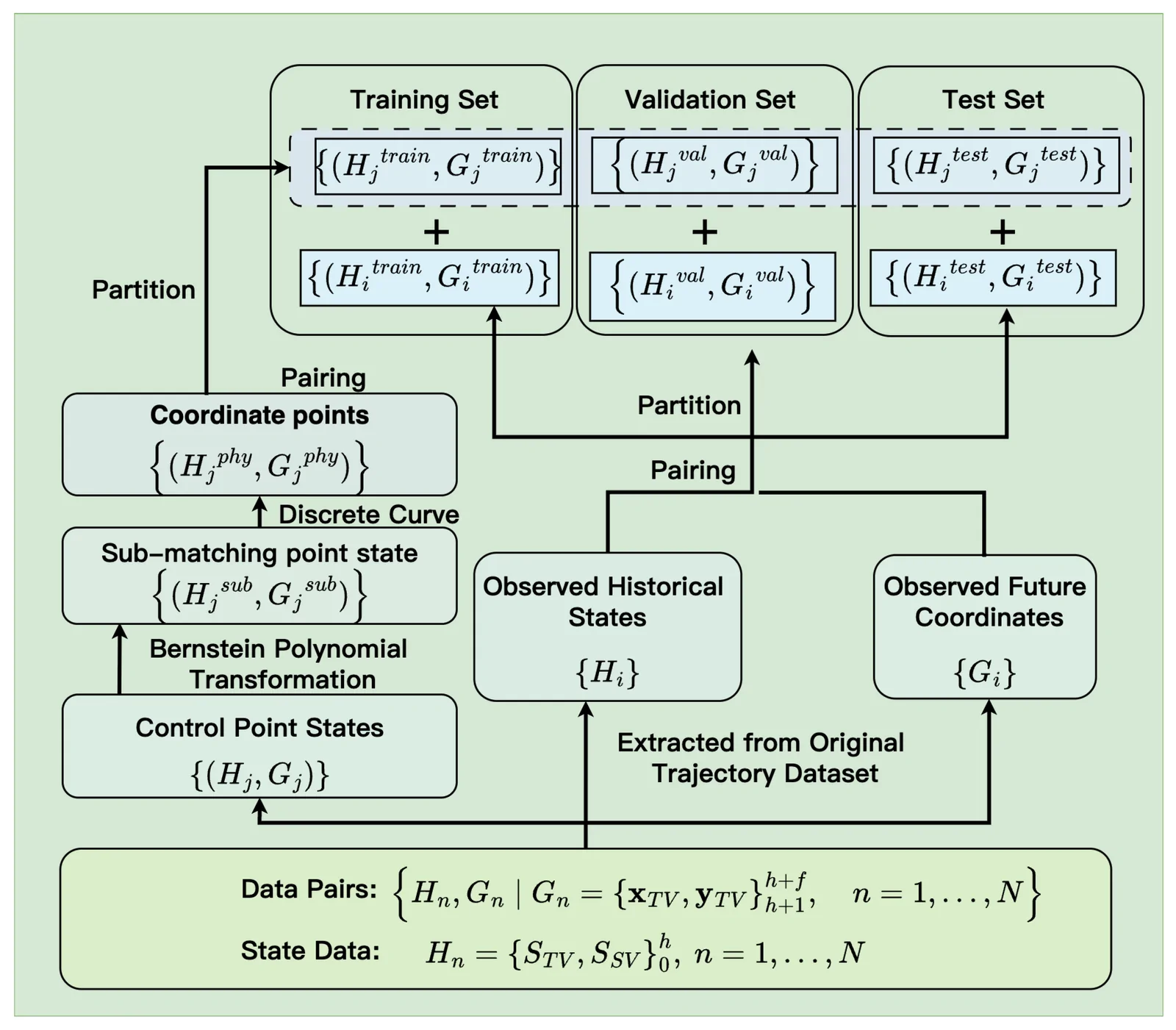
Dataset construction and partitioning strategy. The extended dataset for CMTF includes both conventional observation pairs and additional control-point annotation pairs, and is partitioned into training, validation, and test sets.

Furthermore, as illustrated in Figure 2, the dataset is partitioned into training, validation, and test subsets.

This subsection first defines the historical input and the future label of a sample and then explains how control-point annotations are attached to the same data sample. The historical features of the TV and the Surrounding Vehicles (SV) constitute the observation input of the model:

(2) $$H_{n}={\left\{S_{TV},S_{SV}\right\}}_{0}^{h},\;n=1,\ldots ,N$$

where *N* denotes the total number of samples. The label corresponds to the ground-truth trajectory of the TV:

(3) $$G_{n}={\left\{x_{TV},y_{TV}\right\}}_{h+1}^{h+f},\;n=1,\ldots ,N$$

For conventional data-driven prediction, the input and the label form observation sample pairs. The following equations specify the *i*-th sample:

(4) $$H_{i}={\left\{S_{TV},S_{SV}\right\}}_{0}^{h},\;i=1,\ldots ,N$$

(5) $$G_{i}={\left\{x_{TV},y_{TV}\right\}}_{h+1}^{h+f},\;i=1,\ldots ,N$$

where *N* denotes the number of observation samples.

Beyond these observation pairs, CMTF further constructs control-point annotation pairs. These pairs are obtained through Bernstein polynomial transformation. The historical states remain identical to those of the observation input, whereas the future positions are replaced by future control-point coordinates:

(6) $$H_{j}={\left\{S_{TV}^{phy},S_{SV}^{phy}\right\}}_{0}^{h},\;j=1,\ldots ,N$$

(7) $$G_{j}={\left\{c_{TV}^{x},c_{TV}^{y}\right\}}_{h+1}^{h+f},\;j=1,\ldots ,N$$

Two categories of control points must be clearly distinguished. Historical control points are fitted from the observable historical trajectory and can be constructed during training, validation, and inference. Future control points are fitted from the future ground-truth trajectory. They act as supervision labels only in the training stage, serve solely for offline loss computation or model selection in the validation stage, and are never used as inference inputs. To avoid notational ambiguity, the control points fitted from the historical observed positions $X_{hist}={\left\{x_{TV}\right\}}_{0}^{h}$ are denoted as $C_{hist}=B\left(X_{hist}\right)$, and the control points fitted from the future ground-truth positions $Y_{gt}={\left\{x_{TV},y_{TV}\right\}}_{h+1}^{h+f}$ are denoted as $C_{fut}^{\mathrm{gt}}=B\left(Y_{gt}\right)$. The former are resampled through Bézier interpolation to form $C_{in}$, which constitutes part of the model input. The latter do not enter the model input and are used exclusively to train the control-point prediction head and the geometric consistency loss.

#### 3.2.2. Control Point Integration with Trajectory Fusion

Based on the aforementioned Bernstein polynomial method for converting trajectory points into control points, the control-point encoding logic is systematically integrated from multiple perspectives, including data flow integration, module structure modification, control-point embedding strategies, and interaction mechanisms.

Typically, data-driven vehicle trajectory prediction methods [12,13,39] focus on extracting information from target vehicle features through deep learning networks to generate reliable and accurate future trajectories. These ego-vehicle features fuse semantic and spatiotemporal information transmitted from upstream modules through various network structures. In this work, a Collocation-enhanced Motion Transformer model, CMTR, is proposed as an improved control-point prediction module built upon the Multi-Modal Trajectory Prediction via Motion Transformer model, MTR [12]. CMTR comprises a Transformer encoder for scene context modeling and a Transformer decoder based on motion query pairs, which together constitute the core innovations of the MTR framework and serve as the data-driven components in CMTR. To ensure that the predicted trajectories satisfy the out-of-bound constraint, which keeps generated trajectories within road boundaries, and the repulsion constraint, which prevents collisions between trajectories, CMTR introduces an additional auxiliary prediction head for control-point semantic features on top of the original MTR architecture and models dynamic vehicle interactions across temporal and spatial dimensions through cross-attention. The input thereby incorporates structured geometric constraint information, which strengthens the ability of the model to capture geometrically consistent trajectories.

**Figure 3:**
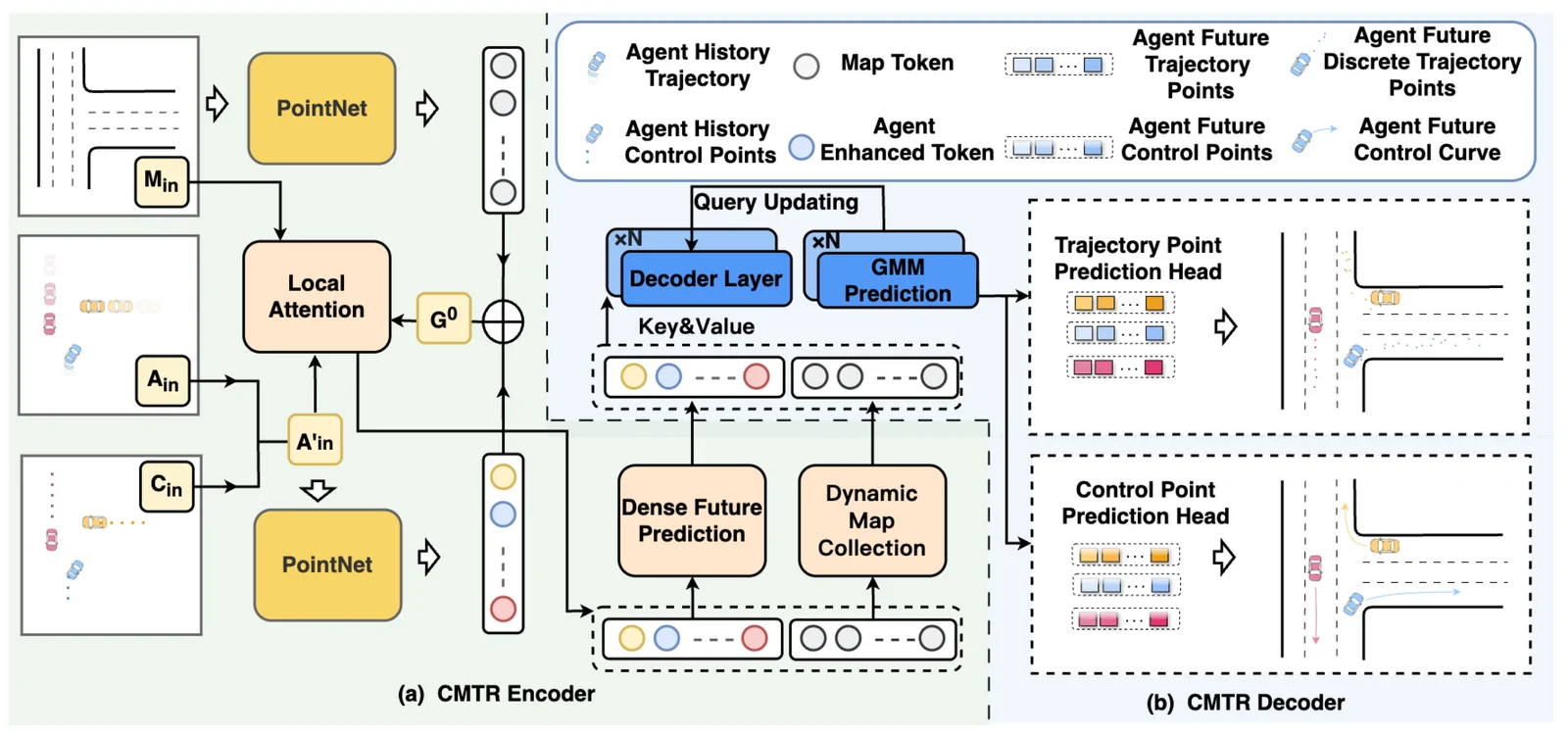
CMTR architecture and control-point integration mechanism.

CMTR Encoder: The encoder augments the vectorized local self-attention Transformer structure of MTR with structured trajectory inputs. As shown in Figure 3a, the model input comprises the historical states $A_{in}$, the multi-segment road map $M_{in}$, and $C_{in}$, which is fitted from the observed historical trajectory and resampled. Here, $C_{in}$ corresponds to the historical control points $C_{hist}$ rather than to the control points fitted from the future ground-truth trajectory. The control point set *C* represents the Bézier curve control vertices required for trajectory fitting. These control points are treated as explicit encodings of the geometric structure information of the trajectory and are integrated into the scene context modeling process, thereby enabling geometrically consistent structured modeling.

However, since the target model samples trajectory points with a Gaussian Mixture Model (GMM), the historical control points do not possess dense temporal information, and directly feeding control points into the deep network makes feature alignment with the historical trajectory states difficult. $C_{in}$ is therefore introduced. The Bézier curve fitted from the historical control points $C_{hist}$ is uniformly sampled over the interval (0,T) to yield a discrete point sequence consistent with the number of observed trajectory frames, which serves as the structured input. In this manner, multi-modal trajectory modeling, uncertainty quantification, and interpretability are preserved, and the input is augmented with historical geometric priors. The process is as follows:

(8) $$C_{in}=\left\{B\left(t_{i};C_{hist}\right)|t_{i}=\frac{i}{T-1},\;i=0,1,\ldots ,T-1\right\}$$

where B(t;C) denotes the Bézier curve defined by the control points *C*, and *T* is the number of observation frames. Subsequently, these controlled discrete points are concatenated with the historical states of the ego vehicle $A_{in}$ to form the enhanced input $A_{in}^{\prime }$:

(9) $$A_{in}^{\prime }=\mathrm{concat}\left(A_{in},C_{in}\right)$$

Although both $A_{in}$ and $C_{in}$ are eventually provided to the model as discrete point sequences, they differ fundamentally in information type and geometric semantics. Specifically, $A_{in}$ represents an observed time series contaminated by measurement noise, whereas $C_{in}$ corresponds to a structured and denoised geometric sequence. Although $C_{in}$ is likewise discretized to align with $A_{in}$ at the feature level, its overall shape is determined by the historical control points $C_{hist}$, thereby explicitly encoding global geometric constraints.

As an explicit representation of structured geometric priors, $C_{in}$ is integrated into the scene context modeling process, which enhances the ability of the model to capture the global structure of trajectories and guides the encoder to focus on structurally salient regions that are critical for decision-making rather than on high-frequency noise. By introducing control points as geometric anchors, the proposed formulation explicitly decouples trajectory shape from sampling noise and enables the model to learn more robust motion patterns.

The concatenated features are encoded into feature vectors $A_{p}$ through a Multi-Layer Perceptron (MLP) within PointNet [40]. These enhanced features, together with the map multi-segment features $M_{p}$, form the encoder input $G_{0}$. The local self-attention mechanism employs a *k*-nearest neighbor algorithm κ(·) to select relevant polyline segments and applies Positional Encoding (PE) for weighted feature aggregation. For the *j*-th Transformer encoder layer:

(10) $$\begin{array}{cc} G_{j}= & \;\mathrm{MHA}\left(\begin{array}{cc} Q= & \;G_{j-1}+\mathrm{PE}\left(G_{j-1}\right),\\ K= & \;\kappa \left(G_{j-1}\right)+\mathrm{PE}\left(\kappa \left(G_{j-1}\right)\right),\\ V= & \;\kappa (G_{j-1}) \end{array}\right) \end{array}$$

CMTR Decoder: The MTR decoder decomposes the motion prediction task into the joint optimization of global intention localization and local movement refinement. Each query pair consists of a static intention query and a dynamic search query. Building upon the motion query pair mechanism, the explicit modeling and decoding of geometric control information are further enhanced. As shown in Figure 3b, an auxiliary prediction head is designed on the query content features output by each decoder layer. This head decodes not only the conventional trajectory distributions, such as the GMM parameters, but also directly predicts control points, which enables the model to proactively learn future constraint information and to integrate it into the trajectory prediction process in an end-to-end manner.

The intention queries $Q_{I}$ are obtained by clustering the ground-truth trajectory endpoints with *k*-means and then passing the position encodings through an MLP:

(11) $$Q_{I}=\mathrm{MLP}\left(\mathrm{PE}\left(I\right)\right)$$

Self-attention between $Q_{I}$ and the previous layer $C_{sa}^{j-1}$ yields the updated $C_{sa}^{j}$:

(12) $$C_{sa}^{j}=\mathrm{MHA}\left(Q_{I}=C_{sa}^{j-1}+Q_{I},\;K_{I}=C_{sa}^{j-1}+Q_{I},\;V_{I}=C_{sa}^{j-1}\right)$$

The dynamic search query $Q_{S}^{j}$ is updated in each decoder layer based on the endpoints of the currently predicted trajectories:

(13) $$C_{A}^{j}=\mathrm{MHA}\left(\left[C_{sa}^{j},Q_{S}^{j}\right],\;\left[A,{\mathrm{PE}}_{A}\right],\;A\right)$$

(14) $$C_{M}^{j}=\mathrm{MHA}\left(\left[C_{sa}^{j},Q_{S}^{j}\right],\;\left[\alpha \left(M\right),{\mathrm{PE}}_{\alpha (M)}\right],\;\alpha \left(M\right)\right)$$

where $Q_{S}^{j}$ tracks the latest predicted trajectory endpoint of each intention and α(M) denotes the set of polyline segments collected along the predicted trajectory.

Finally, the output of the *j*-th decoder layer is given by

(15) $$C^{j}=\mathrm{MLP}\left(\left[C_{A}^{j},C_{M}^{j}\right]\right)$$

The decoder content features $C^{j}$ are passed through a multi-task regression head Head(·) to predict the confidence of each mode and the future state parameters containing control information, and to update $Q_{S}^{j+1}$:

(16) $$\left[Z_{GMM},\;P_{score}\right]=\mathrm{Head}\left(C^{j}\right)$$

The complete inference procedure is as follows. First, the historical states *H* of the target and surrounding vehicles and the map features *M* are provided as input, and $C_{hist}=B\left(X_{hist}\right)$ is fitted solely from the historical position sequence observable for the target vehicle. Second, the Bézier curve corresponding to the historical control points is uniformly sampled to obtain $C_{in}$, which is concatenated with the historical state features and the map features before being fed into the encoder. Subsequently, the decoder generates multi-modal motion queries based on the scene context, and the regression head outputs the future trajectory distribution parameters $Z_{GMM}$, the mode confidence scores $P_{score}$, and optionally the predicted future control points $C_{fut}^{\mathrm{pred}}$. Finally, the future trajectories and their probabilities are decoded from the GMM parameters and the mode confidences as the inference output. During inference, the future ground-truth trajectory $Y_{gt}$, the future ground-truth control points $C_{fut}^{\mathrm{gt}}$, and the reference trajectory constructed from them are never accessed, and the geometric consistency loss is not computed. $C_{fut}^{\mathrm{pred}}$ is an auxiliary output of the model and is not fed back as an input to the current prediction process.

#### 3.2.3. Reconstruction Loss

In addition to the base loss, we further impose a geometric-consistency regularizer on the predicted outputs so that their geometric quality and structural expressiveness remain stable in complex scenarios: each predicted trajectory is compared against the curve fitted from control points, and the resulting penalty steers the network toward structurally coherent outputs. Within this design, the continuous curve produced by the Bernstein parameterization acts as the structural reference. The remainder of this section presents the realization of this regularizer, covering the differentiable reconstruction of Bézier curves, the construction of reference trajectories from ground-truth control points, and the formulation of the combined training objective.

In this mechanism, the future ground-truth control points $C_{fut}^{\mathrm{gt}}=\left\{c_{0},\ldots ,c_{n}\right\}$ are obtained from the control-point dataset constructed in Section 3.2.1, and the continuous reference trajectory ${\hat{Y}}^{\mathrm{Bezier}}$ is generated by interpolating these points with an *n*-th order Bernstein polynomial. Here, $C_{fut}^{\mathrm{gt}}$ is fitted from the future ground-truth trajectory and is available only during training, whereas this variable is not provided at inference. Given a set of predicted control points, the *n*-th order Bernstein polynomial interpolation is likewise applied to generate the predicted continuous trajectory. For any normalized time step $\tau_{t}\in \left[0,1\right]$, the trajectory point position can be expressed as

(17) $${\hat{y}}_{t}^{\mathrm{Bezier}}=\sum_{i=0}^{n}B_{i}^{n}\left(\tau_{t}\right)\cdot c_{i}^{\mathrm{gt}}$$

Simultaneously, the model outputs a set of predicted control points $C^{\mathrm{pred}}$ via the control point prediction head, which are likewise interpolated by the Bernstein polynomial to generate the predicted trajectory ${\hat{Y}}^{\mathrm{pred}}$ for structural alignment with the reference trajectory.

Based on this, a geometric consistency loss $L_{\mathrm{consistency}}$ is constructed, which measures the mean squared error between the predicted and reference trajectories across all time steps:

(18) $$L_{\mathrm{consistency}}=\frac{1}{NT}\sum_{i=1}^{N}\sum_{t=1}^{T}{∥{\hat{y}}_{i,t}^{\mathrm{pred}}-{\hat{y}}_{i,t}^{\mathrm{Bezier}}∥}_{2}$$

where *N* is the batch size, *T* is the number of prediction time steps, and *i* denotes the sample index.

The above loss is integrated into the final training objective $L_{\mathrm{total}}$, which includes both the base loss $L_{\mathrm{base}}$ and the geometric consistency loss $L_{\mathrm{consistency}}$:

(19) $$L_{\mathrm{total}}=\left(1-\lambda \right)L_{\mathrm{base}}+\lambda \cdot L_{\mathrm{consistency}}$$

where λ is a tunable weighting coefficient that balances the contributions of the individual loss terms.

**Figure 4:**
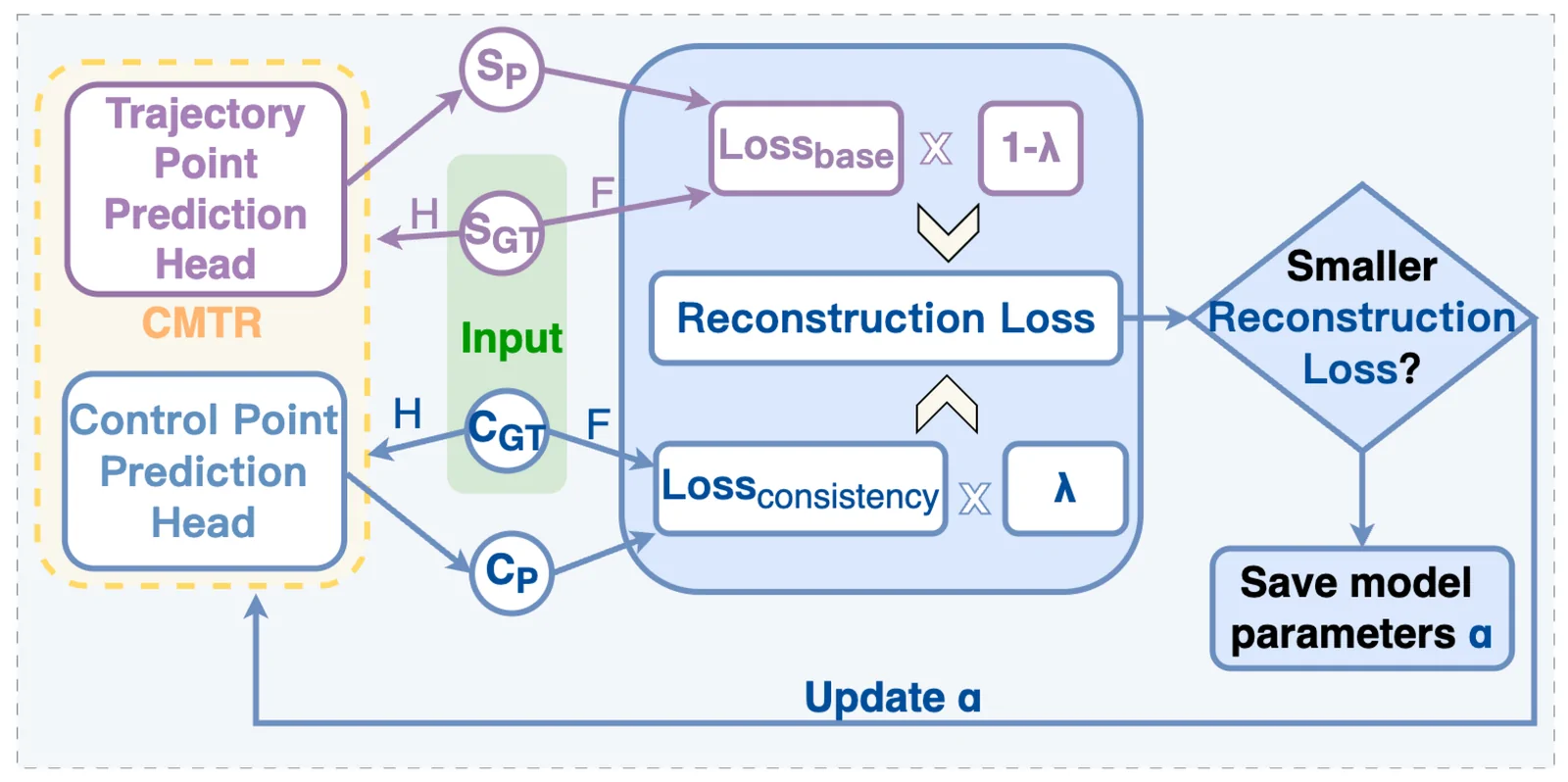
CMTR loss reconstruction mechanism. The model utilizes the collaborative state of historical observations and control points as input and, through the differentiable Bézier interpolation, structurally supervises the prediction of control points and generated trajectories.

As illustrated in Figure 4, during CMTR training, the collaborative state composed of the observed historical states $S_{\mathrm{GT}}$ and the historical control points $C_{\mathrm{GT}}$ fitted from historical observations is taken as input. After feature extraction by the encoder, the model predicts both the future trajectory states $S_{P}$ and the future control points $C_{P}$. Through a differentiable Bézier interpolation process, the model generates the predicted trajectory ${\hat{Y}}^{\mathrm{pred}}$, which is compared with the reference trajectory ${\hat{Y}}^{\mathrm{Bezier}}$ constructed from the ground-truth future control points $C_{fut}^{\mathrm{gt}}$ to compute the geometric consistency loss. Structured supervision of the control point prediction is thereby achieved.

## 4. Experiments

Extensive empirical studies are conducted based on the CMTF. For in-domain experiments, three mainstream baseline models are selected for comparison, and CMTF variants of two classical models are further constructed. Subsequently, these two variant models are utilized in cross-domain experiments to systematically investigate the following two core research questions:

1. Can CMTF variants maintain or surpass the performance of the original models in in-domain scenarios?
2. Without increasing the number of training samples, can CMTF variants enhance the OoD generalization capability of the models?

### 4.1. Experimental Setup

#### 4.1.1. Datasets

In this study, quantitative and qualitative domain generalization experiments on the proposed CMTF are conducted using three vehicle trajectory prediction datasets: nuScenes [5], Argoverse2 [6], and WOMD [7]. The detailed split of sample counts for each dataset is presented in Table 2.

- nuScenes: Released by nuTonomy, the autonomous driving division of Aptiv that is now Motional, the nuScenes dataset comprises 1000 driving scenes collected in Boston and Singapore. Each scene lasts 20 s and is annotated at 2 Hz, corresponding to 0.5 s per frame. The dataset is publicly available at https://www.nuscenes.org.
- Argoverse2: Released by Argo AI in collaboration with Carnegie Mellon University in 2023, Argoverse2 is an open-source autonomous-driving dataset containing motion data and high-definition maps from six U.S. cities: Austin, Detroit, Miami, Pittsburgh, Palo Alto, and Washington, D.C. The dataset focuses on interactive driving scenarios and is annotated at 10 Hz. It includes diverse road configurations, such as intersections, highway ramps, and roundabouts, together with high-definition map information. The dataset is publicly available a https://www.argoverse.org/.
- WOMD: Released by Waymo in 2021, WOMD consists of 1150 scenes organized as 20-s sequences sampled at 10 Hz by multiple sensors. The data were collected in San Francisco, Phoenix, and Mountain View and cover diverse traffic scenarios such as urban streets, highways, and intersections. The dataset can be accessed at https://waymo.com/open/data/motion/ (accessed on 5 October 2026).

**Figure 5:**
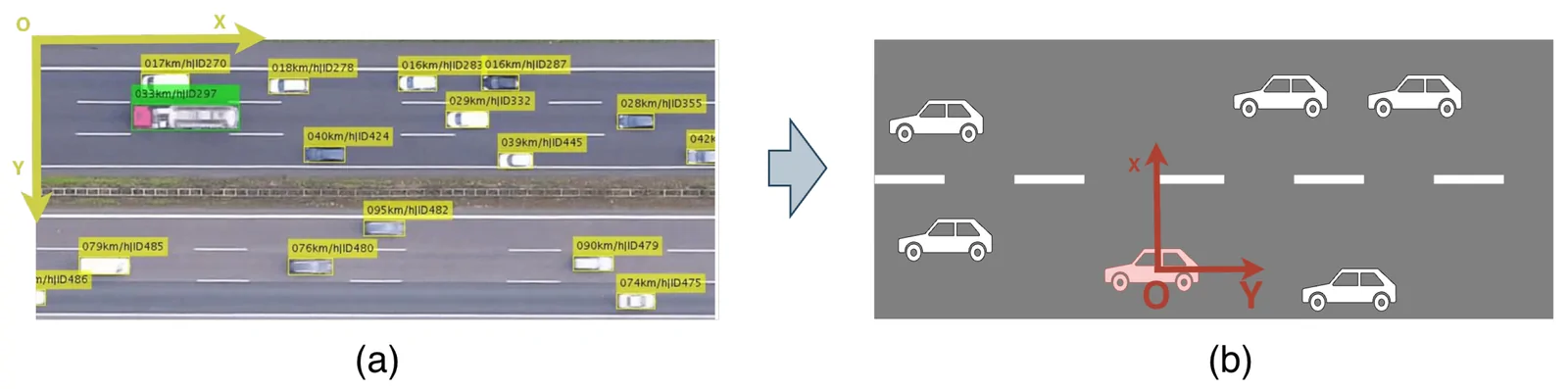
Illustration of coordinate system transformation. (**a**) Scene-level map-based coordinates. (**b**) Agent-centric, vectorized coordinates used in CMTF.

Given the differences among the three datasets in native format, sampling rate, and scene composition, scenario serialization and preprocessing are completed through ScenarioNet [41], an open-source platform for large-scale traffic scenario simulation and modeling, prior to any cross-dataset comparison. The specific preprocessing steps are as follows:

1. Target-agent-centric coordinate alignment: The original scene-level coordinates of each dataset are uniformly converted into a target-agent-centric vectorized coordinate system, in which the longitudinal direction of vehicle motion corresponds to the positive y-axis and the lateral direction to the positive x-axis, as illustrated in Figure 5. For example, nuScenes takes the upper-left corner of the scene map as the origin, with the x- and y-axes pointing rightward and downward. This conversion eliminates the systematic offsets introduced by the differing global coordinate conventions of the datasets.
2. Unified state fields: All agent states are standardized into a unified set of fields, including position, heading angle, velocity, and validity flags.
3. Temporal resolution alignment and fixed time windows: nuScenes is annotated at 2 Hz, whereas Argoverse2 and WOMD are annotated at 10 Hz. To unify the temporal resolution, the nuScenes trajectories are resampled to 10 Hz through linear interpolation, corresponding to 0.1 s per frame, so that the three datasets share the same time step. Fixed windows are then sampled with 21 historical frames covering 2.1 s and 60 prediction frames spanning 6.0 s. Meanwhile, the influence of the height and dimensions of the predicted agent on the trajectory is assumed to be negligible. The ‘z_axis’ and ‘size’ fields are therefore masked, and all data are converted from float64 to float32 to reduce storage.
4. Unified map elements: Vectorized map elements are consolidated into six categories of polyline inputs: lanes, stop signs, road edges, road markings, crosswalks, and speed bumps.

Two aspects of this protocol merit emphasis. First, the above processing unifies only the data format and the task protocol, that is, an alignment at the representation level. It does not eliminate the distributional differences among the three datasets in geographic regions, for example, Boston and Singapore versus multiple US cities, road topology, traffic density, and scene composition. These residual distributional differences constitute precisely the distribution shift that the OoD evaluation is intended to measure, and the cross-dataset tests therefore remain meaningful. Second, the unified protocol builds upon the community-established tool ScenarioNet [41]. Its role is to ensure fairness in cross-dataset comparisons so that performance differences can be attributed to the models and their supervision mechanisms rather than to discrepancies in data format.

#### 4.1.2. Baselines

To demonstrate the effectiveness of CMTF, comparative studies are conducted using Wayformer as a baseline reference, while the CMTF module is integrated into the other two state-of-the-art models, MTR and AutoBot. These models represent complementary perspectives on agent trajectory prediction, enabling a focused evaluation of the performance gains attributable to the proposed CMTF module.

- MTR [12] introduces a motion Transformer framework that integrates global intention localization with local movement refinement, thereby improving the accuracy of multi-modal trajectory prediction.
- AutoBot [13] is based on a set Transformer with latent variables and enables joint prediction for multiple agents, improving interaction modeling capabilities.
- Wayformer [39] proposes an efficient attention-based network, offering a lightweight prediction method that optimizes computational efficiency while maintaining predictive performance.

In addition, to demonstrate the effectiveness of the proposed supervision mechanism, additional model variants are further designed and evaluated. The extended parameter differences and computational efficiency details are reported in Table 3 and Table 4.

#### 4.1.3. Evaluation Metrics

For ID evaluation, the official benchmark metrics are adopted, including minimum Average Displacement Error (minADE), minimum Final Displacement Error (minFDE), Miss Rate (MR), and Brier-minimum Final Displacement Error (brier-minFDE).

minADE: The minADE metric measures the mean Euclidean distance between the predicted and ground-truth trajectory points at each time step. Let $y_{n,t}$ denote the ground-truth position of the *n*-th trajectory at time step *t*, and ${\bar{y}}_{n,t}$ denote the corresponding predicted position. The minADE is defined as follows:

(20) $$\mathrm{minADE}=\frac{1}{T\times N}\sum_{t=1}^{T}\sum_{n=1}^{N}{∥{\bar{y}}_{n,t}-y_{n,t}∥}_{2}$$

where *T* is the total number of time steps, *N* is the total number of trajectories, and ${∥\cdot ∥}_{2}$ denotes the Euclidean distance between the predicted and ground-truth positions.

minFDE: The minFDE metric emphasizes the accuracy of the predicted trajectory at the final time step. It specifically measures the Euclidean distance between the predicted and ground-truth final positions, providing a focused evaluation of long-term forecasting and final decision accuracy. The minFDE is defined as

(21) $$\mathrm{minFDE}=\frac{1}{N}\sum_{n=1}^{N}\min{∥{\bar{y}}_{n,T}-y_{n,T}∥}_{2}$$

where minFDE solely considers the Euclidean distance between the predicted and true endpoints, offering a precise metric for assessing the accuracy and rationality of long-term predictions and final decisions.

Miss Rate: The Miss Rate evaluates whether the predicted trajectory falls within a specified threshold of the ground-truth trajectory. If the deviation exceeds the threshold Φ, the prediction is counted as a miss. The MR is calculated as

(22) $$\mathrm{MR}=1-\frac{\sum_{k=1}^{K}m\left({\bar{y}}_{k},y_{k}\right)}{N}$$

where $m({\bar{y}}_{k},y_{k})$ denotes the event that both the x- and y-directional errors between the predicted point and the ground-truth point are below their respective dynamic thresholds.

brier-minFDE: This metric combines the Final Displacement Error with the Brier Score, weighted by prediction probabilities, to jointly evaluate prediction accuracy and uncertainty. The brier-minFDE is defined as

(23) $$\mathrm{brier-minFDE}=\mathrm{minFDE}+{\left(1-\frac{\min p_{n}}{\sum_{n=1}^{K}p_{n}}\right)}^{2}$$

where $p_{n}$ represents the predicted probability that the *n*-th trajectory matches the ground truth.

For OoD evaluation, in addition to the above metrics, ΔminADE, ΔminFDE, ΔMR, and Δbrier-minFDE are further computed to measure the performance degradation under distribution shift. A smaller value indicates that the performance of the model on OoD data is closer to that on ID data, reflecting a stronger capacity to learn generalizable patterns.

#### 4.1.4. Implementation Details

##### Coordinate System Transformation

Taking the original nuScenes dataset as an example, the coordinate system origin is defined at the upper-left corner of the scene map, as illustrated in Figure 5a. The positive *x*-axis and *y*-axis extend rightward and downward from the origin, respectively. CMTF, in contrast, primarily utilizes vectorized, agent-centric data as input. The data processing pipeline is designed to convert the scene-level raw data into this reformatted structure, in which the vehicle’s longitudinal and lateral directions correspond to the positive *y*-axis and *x*-axis, respectively, as shown in Figure 5b.

##### Experimental Settings

The models are trained on two NVIDIA A100 GPUs. The batch size for MTR, AutoBot, and Wayformer is set to 128. Training is conducted using the AdamW optimizer with an initial learning rate of 0.0001, and the learning rate is decayed by a factor of 0.5 every 2 epochs starting from epoch 20. The weight decay is set to 0.01. The model checkpoint with the best min-brierFDE on the validation set is selected for evaluation. For all three datasets, a unified preprocessing pipeline is applied using ScenarioNet [41], an open-source platform for large-scale traffic scenario simulation and modeling, following the dataset splits shown in Table 2.

For the trajectory prediction task, it is assumed that the size and height of the predicted agents have a negligible impact on the trajectory. The ‘z_axis’ and ‘size’ parameters are therefore masked, and all data are converted from float64 to float32 to save disk space. Additionally, the line types included in the network input are lanes, stop signs, road edges, road markings, crosswalks, and speed bumps. Hyperparameter details are listed in Table 5.

### 4.2. Main Results

#### 4.2.1. ID Test

The proposed method is evaluated using multiple backbone models, consistent with the experimental protocols of related studies. The best ID test results for all models are reported in Table 6 and Table 7. Although ID test performance is not the primary focus of this research, competitive or even state-of-the-art results are achieved by CMTF under specific values of λ.

On the nuScenes and Argoverse2 datasets, CMTF yields consistent improvements in the reported single-run results. Specifically, on nuScenes, CMTF reduces the minFDE6 of MTR by 7.06% and the MR by 8.7%. The improvements for AutoBot are more pronounced, with minFDE6 decreased by 12.97% and MR reduced by 17.65%. On Argoverse2, despite the already strong performance of the baseline models, CMTF further reduces the brier-minFDE of MTR by 4.64% and the minFDE6 by 5.43%.

#### 4.2.2. OoD Test

To evaluate the generalization capability of the proposed framework, nuScenes and Argoverse2 serve as training source domains, while nuScenes, Argoverse2, and WOMD serve as target test domains, and WOMD is used exclusively as a target test domain. The OoD results mainly compare the two backbones, MTR and AutoBot, together with their CMTF variants, and the complete results are presented in Table 8 and Table 9. This cross-evaluation over three datasets and two backbones is designed to examine the transferability of the method under different data distributions.

An analysis of the tabulated data reveals a noteworthy phenomenon. Certain model configurations perform better in OoD testing than in their ID tests. Specifically, for the MTR model trained on nuScenes, the brier-minFDE on Argoverse2 reaches 2.58, which is lower than the ID test result of 3.17 on nuScenes. Similarly, the same metric for the AutoBot model improves from 4.39 to 2.37.

A plausible explanation for this seemingly paradoxical behavior lies in the mismatch in scenario composition between the two datasets: nuScenes spans a broader spectrum of scene types and contains a higher proportion of challenging edge cases, whereas the Argoverse2 test split appears to consist largely of relatively regular, well-structured traffic layouts. Because training on nuScenes already exposes the models to highly diverse maneuver patterns, the feature repertoire they acquire covers most scene categories present in Argoverse2, which in turn eases the transfer of learned knowledge.

Further analysis reveals differences in the adaptability of the baseline models across OoD scenarios. For instance, AutoBot exhibits favorable numerical results in the WOMD test scenarios, with a brier-minFDE of 4.69 that is lower than that of MTR. With the integration of CMTF, the reported model configurations yield lower metrics in OoD testing. Among them, AutoBot achieves the largest reduction in brier-minFDE, at 16.03%, when transferring from nuScenes to Argoverse2.

**Figure 6:**
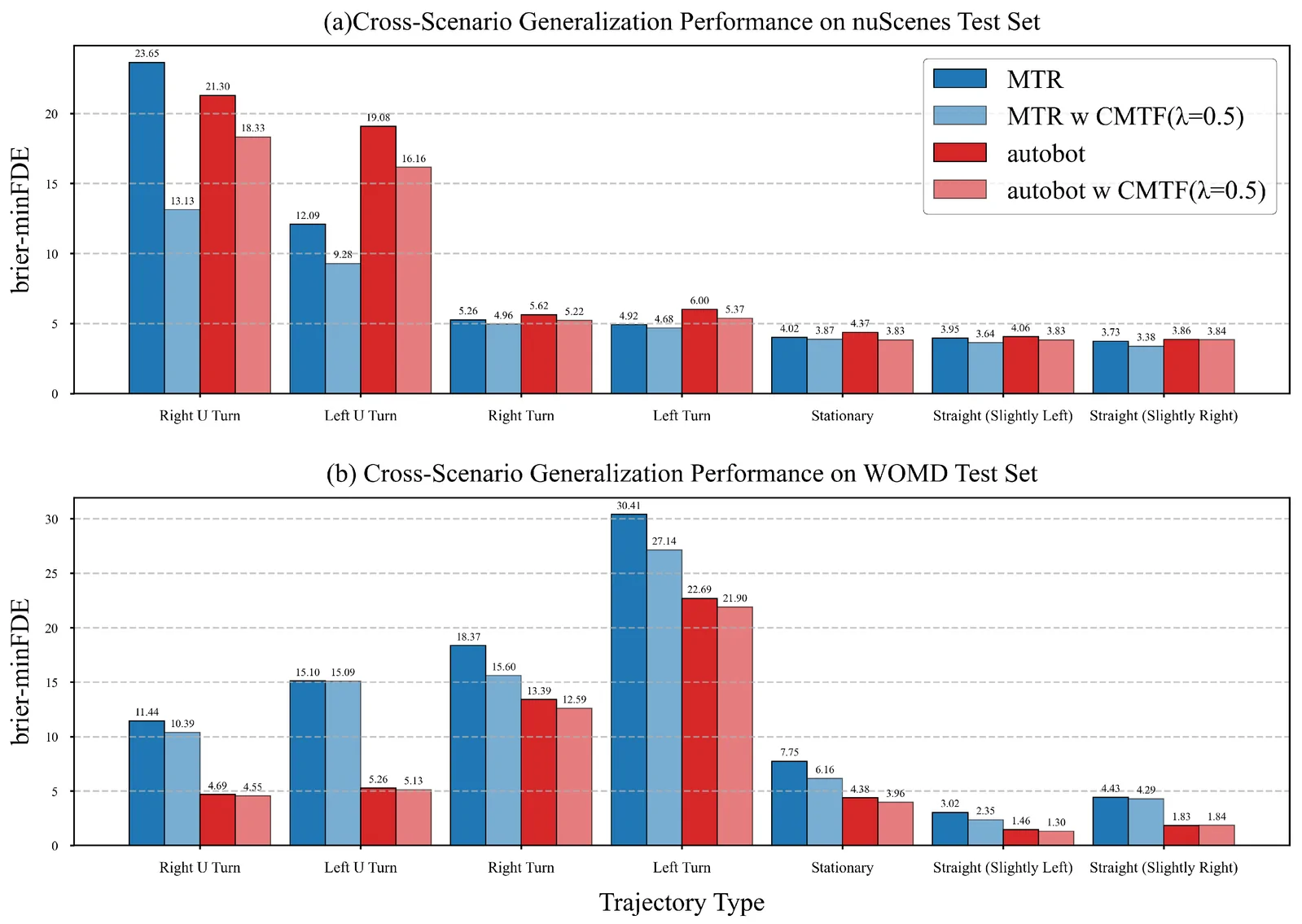
Cross-scenario generalization experiments are conducted across diverse traffic scenarios, with rows representing model test sets. The Brier-minFDE of models trained on Argoverse2 is reported.

As shown in Figure 6, CMTF yields considerable numerical improvements on several complex trajectory types in the cross-scenario evaluations. For instance, on the nuScenes test set, the brier-minFDE of MTR in the Right U Turn scenario decreases from 23.65 to 13.13, a reduction of 44.5%. On the WOMD test set, the error of AutoBot in the Stationary scenario decreases from 7.75 to 6.16, while the lowest value of 1.30 is attained in the Slightly Left case. Overall, CMTF exhibits favorable cross-scenario numerical trends on challenging maneuvers such as turns and U-turns while remaining relatively stable on simpler trajectories.

**Figure 7:**
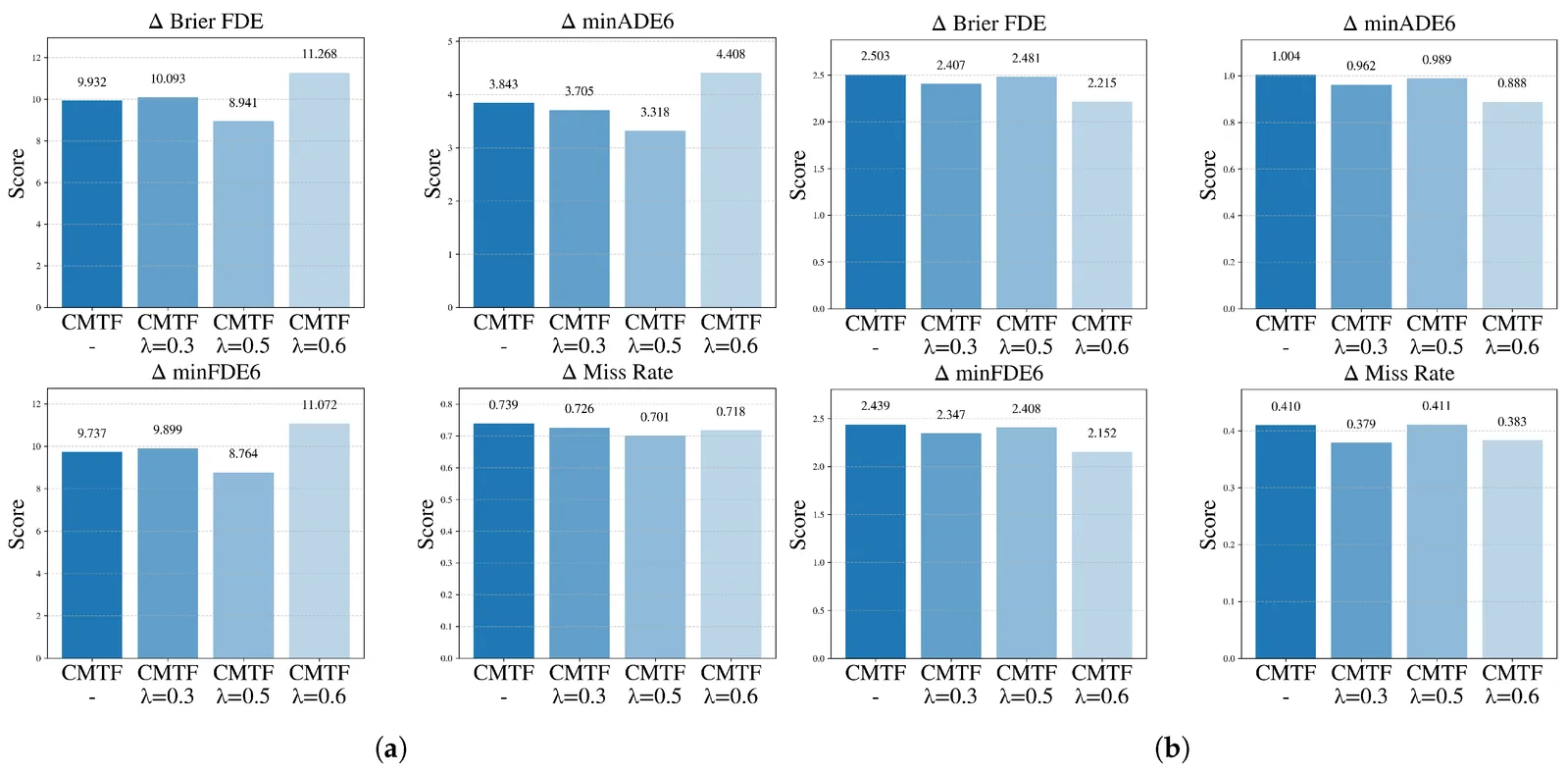

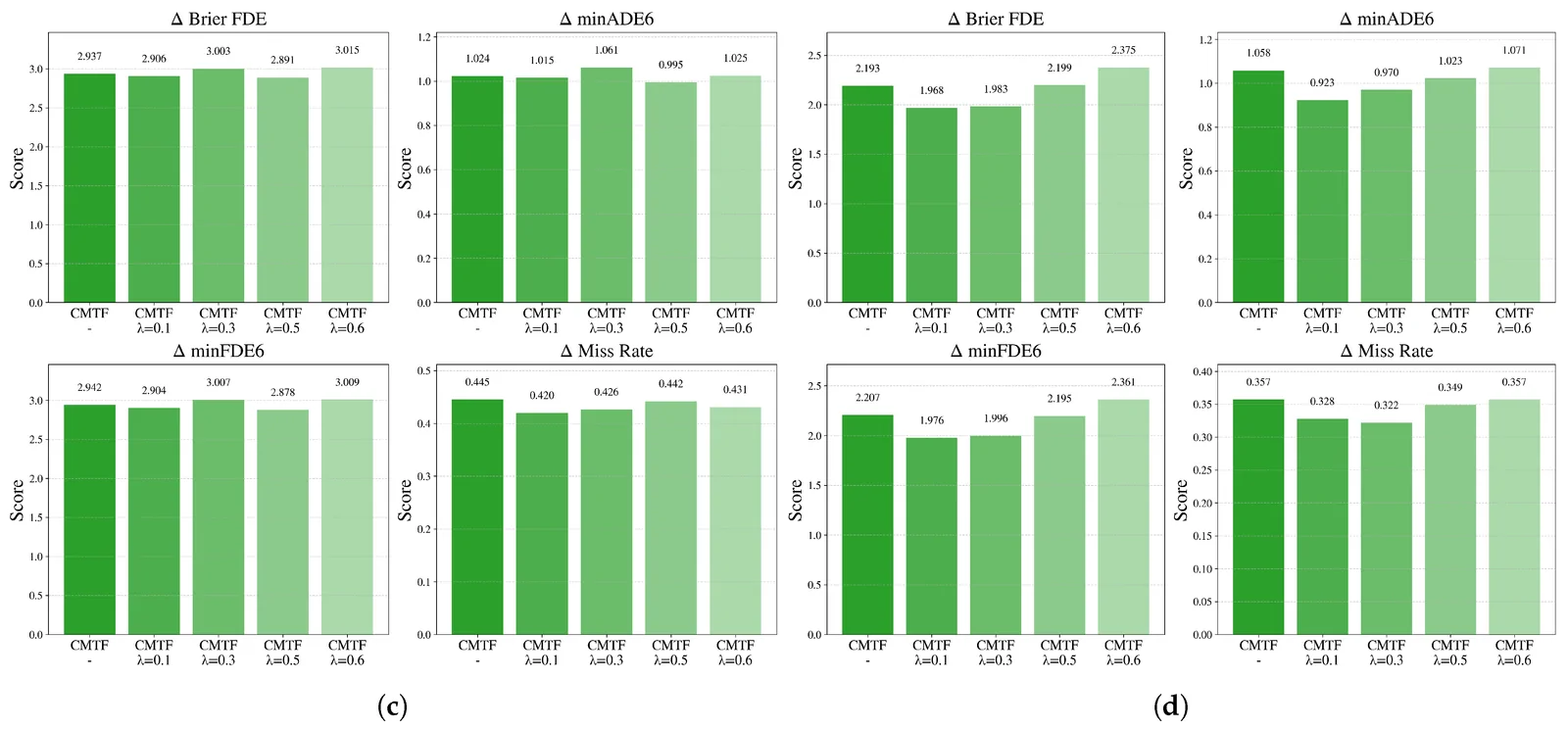
Growth rates of key metrics for models trained on Argoverse2 and evaluated on nuScenes and WOMD, under different geometric consistency loss weights λ in the CMTF. (**a**) Results of MTR model equipped with CMTF module on nuScenes test set. (**b**) Results of MTR model equipped with CMTF module on WOMD test set. (**c**) Results of AutoBot model equipped with CMTF module on nuScenes test set. (**d**) Results of AutoBot model equipped with CMTF module on WOMD test set. “CMTF”—denotes the baseline models without the CMTF module.

The central question of this investigation is whether the ID performance of CMTF can be maintained under OoD testing, thereby examining the cross-domain effect of the structured representation. To this end, the performance degradation of each model under distribution shift is further evaluated, with the results reported in Figure 7. In this context, the increase in the prediction metrics serves as an indicator of model robustness. The optimal configuration, MTR with CMTF at λ=0.5, is lower than the original MTR across all reported metrics, with cross-domain errors reduced by approximately 10%. Although AutoBot already exhibits favorable OoD numerical performance on this task, its MR is further reduced by 5.62% after the integration of CMTF. In addition, on the WOMD test set, CMTF brings considerable numerical reductions for both MTR and AutoBot, with the primary metrics decreasing by approximately 10%. Notably, AutoBot with CMTF at λ=0.1 achieves the best result among the listed configurations.

### 4.3. Ablation Studies

In multi-task learning, balancing the loss contributions from different tasks remains a critical challenge. Table 10 provides both a framework-level switch comparison and a sensitivity analysis of the geometric consistency loss weight, systematically evaluating how different values of λ regulate model performance on the nuScenes and WOMD test sets after training on Argoverse2. The MTR and AutoBot rows marked with a dash denote the original backbones with the entire CMTF control-point pathway disabled, that is, the CMTF-off configuration at the implementation level. In the implementation of this paper, this configuration is produced by a framework switch at λ=0. Besides setting the geometric consistency loss weight to zero, the structured input is not constructed and the auxiliary control-point prediction branch is deactivated. The remaining rows enable the complete closed loop with varying positive values of λ. Consequently, the comparison between the dash rows and the CMTF rows assesses the overall effect of the closed-loop mechanism, whereas the comparison among different positive λ configurations analyzes the strength of the structural supervision.

For the MTR model, the configuration with λ=0.6 achieves the best results on the nuScenes test set, where the brier-minFDE decreases from the baseline value of 4.009 to 3.757, an improvement of 6.29%, and the minFDE6 decreases from the baseline value of 3.361 to 3.108, an improvement of 7.53%. However, the same configuration performs worst on the WOMD test set, where the brier-minFDE rises to 12.810, an increase of 11.99% over the baseline value of 11.438, indicating that the optimal value of λ is task-dependent. MTR attains its best WOMD performance at λ=0.5, where the brier-minFDE decreases to 10.386, an improvement of 9.20% over the baseline.

For the AutoBot model, λ=0.5 achieves the optimal results on both test domains, where the brier-minFDE on nuScenes decreases from the baseline value of 3.945 to 3.683, an improvement of 6.64%, and that on WOMD decreases from the baseline value of 4.689 to 4.555, an improvement of 2.86%. In contrast, λ=0.6 degrades to 4.094 on nuScenes, which is even below the baseline level.

The sensitivity analysis in Table 10 was used to identify a representative value of λ for the present study. The analysis was conducted after the main model configurations had been defined, and was not used to claim a target-domain-independent hyperparameter-selection procedure. Among the tested values, λ=0.5 provides the most balanced behavior across the evaluated backbone models and test domains. Specifically, it improves over the CMTF-free baseline on both reported test domains for both MTR and AutoBot. For MTR, the brier-minFDE values on nuScenes and WOMD are 3.926 and 10.386, respectively, compared with baseline values of 4.009 and 11.438. For AutoBot, the corresponding values are 3.683 and 4.555, compared with baseline values of 3.945 and 4.689. In addition, λ=0.5 is either the best-performing value or within 0.169 of the best value across the four model-domain combinations.

We acknowledge that using the observed sensitivity results from the evaluated domains to select a representative value may introduce a degree of test-domain-informed model selection. Therefore, the present study should not be interpreted as providing a strictly target-domain-independent hyperparameter-selection protocol. Instead, λ=0.5 is used as a representative compromise for reporting the main comparisons. Future work will investigate validation-only or source-domain-only selection procedures, nested evaluation protocols, and adaptive constraint-weight strategies that do not use target-test performance.

Meanwhile, across the four combinations of the model and test domain, λ=0.5 is either optimal or within 0.169 of the best result. More importantly, under the OoD protocol the target domain is unknown during training. Tuning λ individually for each target domain would introduce target-domain information leakage and undermine the validity of the OoD evaluation. The appropriate selection criterion for λ is therefore cross-target-domain robustness rather than single-point optimality. Given that this study aims to validate the effectiveness of structured representations rather than adaptive optimization, λ=0.5 is selected as the representative weight, as it combines consistent gains on both domains with robustness across models, and adaptive λ selection strategies oriented to the degree of domain shift are left to future work.

### 4.4. Qualitative Analysis

**Figure 8:**
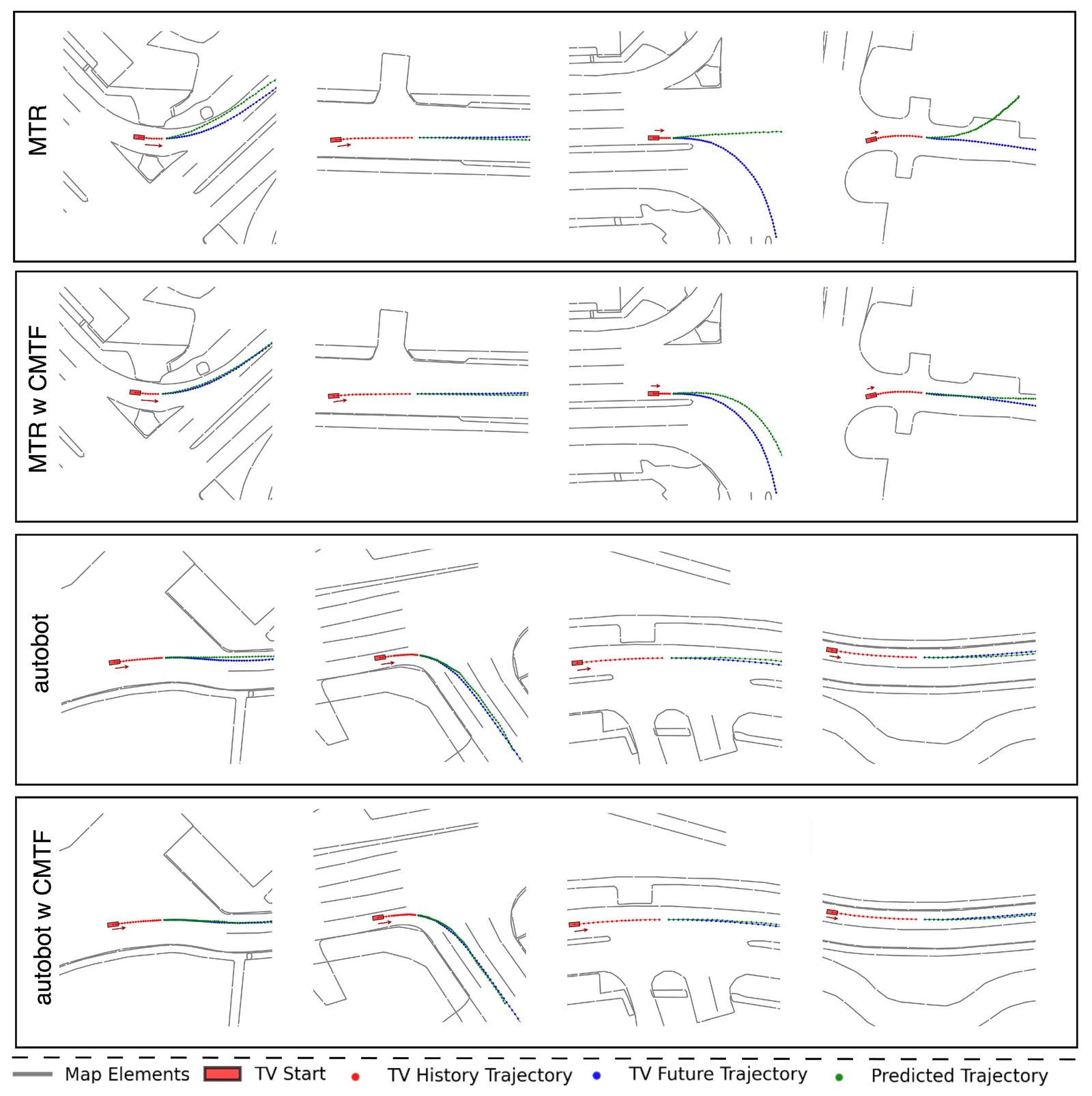
Cross-domain trajectory prediction visualization comparison on the nuScenes test set. For each scenario, predictions from baseline models and their CMTF variants are shown. CMTF-enhanced models demonstrate improved geometric consistency and trend alignment, while baseline models may produce implausible sharp turns and discontinuities, particularly in challenging scenes.

This section presents a qualitative comparison of the predicted trajectories of the baseline models and their CMTF-enhanced variants, both trained on the Argoverse2 dataset and evaluated on the nuScenes test set under cross-domain generalization settings. As illustrated in Figure 8, across the displayed scenarios, the CMTF variants exhibit fairly consistent numerical and shape improvements over the baseline models, particularly in terms of geometric structure and directional trend alignment. The baseline models tend to produce trajectories with unrealistic sharp turns and discontinuities, especially in complex traffic environments. In contrast, the models equipped with CMTF generate smoother and structurally more reasonable trajectories that better conform to actual traffic flow patterns and lane geometries.

## 5. Conclusions

A general control-point guided trajectory prediction framework is proposed to address two critical bottlenecks of existing methods, namely weak OoD generalization and the lack of geometric consistency. By introducing a spatially semantic control-point representation and integrating Bernstein polynomial parameterization with a geometric consistency loss, CMTF enables structured trajectory modeling and end-to-end constrained optimization. With respect to methodological positioning, Bernstein polynomial parameterization and least-squares fitting are both classical techniques. The contribution of this work lies in the closed-loop control-point supervision mechanism built upon them, the joint design of structured pseudo-trajectory inputs, an auxiliary control-point prediction head, and a geometric consistency loss, together with the consistent improvement in OoD generalization achieved without increasing the amount of training data. Owing to its pluggable nature, CMTF can be seamlessly integrated into mainstream prediction models and exhibits a trend of OoD performance improvement in the reported configurations on nuScenes, Argoverse2, and WOMD. It should be noted that the conclusions of this paper should be interpreted as descriptive trends across three datasets and two primary backbones rather than statistically tested population-level conclusions. Future work will further assess result stability through multi-seed repetitions, error distribution visualization, and paired statistical tests.

Regarding hyperparameter sensitivity, the optimal value of the geometric consistency loss weight λ varies with the backbone model and the target domain. Future research will focus on more flexible trajectory parameterization methods and adaptive constraint mechanisms, including exploring adaptive-order spline curves or other parameterization forms that dynamically adjust smoothness according to motion patterns, systematically assessing the performance differences of various curve fitting and kinematic constraints in cross-domain scenarios, and investigating adaptive selection strategies for structural constraint weights to handle varying domain shifts and scene complexities. Regarding applicability to real driving scenarios, the evaluations in this paper are all completed on offline datasets. Future work will integrate CMTF with reinforcement learning and closed-loop control systems, in which control points serve as intermediate decision variables, to advance trajectory prediction from trajectory shape learning toward real-vehicle deployability.

## Figures and Tables

**Table 1:** Notation used throughout this paper.

Symbol	Description
*H*/$A_{in}$	Historical state features of the TV and surrounding vehicles
*M*/$M_{in}$	Map polylines and their encoded features
$C_{hist}$	Historical control points fitted from the observed historical trajectory
$C_{fut}^{\mathrm{gt}}$	Future control points fitted from the future ground-truth trajectory, used only for training supervision
$C_{in}$	Bézier-resampled sequence of $C_{hist}$, serving as the structured input
$A_{in}^{\prime }$	Augmented input obtained by concatenating the historical states with $C_{in}$
$G_{j}$	Scene context features of the *j*-th encoder layer
$Q_{I}$/$Q_{S}^{j}$	Static intention queries/dynamic search queries of the *j*-th layer
$C_{A}^{j}$/$C_{M}^{j}$	Query contexts extracted from agent features/map features
$C^{j}$	Fused query content features of the *j*-th decoder layer
$Z_{GMM}$/$P_{score}$	Distribution parameters of future trajectories/confidence scores of the predicted modes
$C_{fut}^{\mathrm{pred}}$	Future control points output by the auxiliary prediction head
$L_{base}$/$L_{consistency}$	Base prediction loss/geometric consistency loss
λ	Weighting coefficient of the geometric consistency loss

**Table 2:** Number of trajectories in the train, test, and validation splits.

Dataset	Train	Test	Val
Argoverse2	183,333	22,789	22,979
nuScenes	23,145	9041	9041
WOMD	1,881,966	165,521	164,505

**Table 3:** Comparison of AutoBot and AutoBot with CMTF model parameters (in millions).

Model	Agents Encoder	Total Params (M)	Trainable Modules	Avg. Inference Time (ms)	Avg. Throughput (Samples/s)
AutoBot	0.384	6.027	110	26.61	1317.93
w/CMTF	0.640	6.162	117	27.31	1250.73

**Table 4:** Comparison of MTR and MTR with CMTF model parameters (in millions).

Model	Context Encoder (M)	Motion Decoder (M)	Avg. Inference Time (ms)	Avg. Throughput (Samples/s)
MTR	5.1	60.1	63.11	543.85
w/CMTF	5.5	60.6	64.28	525.16

**Table 5:** Details of hyperparameters.

Description	Symbol	Value
MR threshold (m)	Φ	2
Training epochs	Epoch	40
History length (frames)	*H*	21
Prediction length (frames)	*F*	60
Number of modes	*k*	6

**Table 6:** ID evaluation on the nuScenes dataset. The best results are in bold, and the second-best results are underlined. ↓ indicates that lower values are better.

Model	brier_fde ↓	minADE6 ↓	minFDE6 ↓	miss_rate ↓
MTR	3.17	1.13	2.55	0.46
AutoBot	4.39	1.68	3.70	0.68
Wayformer	3.23	**1.09**	2.58	0.46
MTR w/CMTF (λ = 0.6)	**2.97**	1.12	**2.37**	**0.42**
AutoBot w/CMTF (λ = 0.5)	3.90	1.53	3.22	0.56

**Table 7:** ID evaluation on the Argoverse2 dataset. The best results are in bold, and the second-best results are underlined. ↓ indicates that lower values are better.

Model	brier_fde ↓	minADE6 ↓	minFDE6 ↓	miss_rate ↓
MTR	1.51	0.49	0.92	0.09
AutoBot	1.75	0.53	1.05	0.13
Wayformer	1.60	0.51	0.97	0.09
MTR w/CMTF (λ = 0.5)	**1.44**	**0.47**	**0.87**	**0.08**
AutoBot w/CMTF (λ = 0.5)	1.66	0.51	0.97	0.12

**Table 8:** Cross-dataset evaluation comparison. The Brier-minFDE metric is reported. The best results are in bold. Lower values are better.

Evaluation	Model	Training	
		nuScenes	Argoverse2
nuScenes	MTR	3.17	3.93
	MTR w/CMTF (λ = 0.5)	**3.05**	**3.75**
Argoverse2	MTR	2.58	1.51
	MTR w/CMTF (λ = 0.5)	**2.46**	**1.44**
WOMD	MTR	11.89	11.44
	MTR w/CMTF (λ = 0.5)	**11.54**	**10.38**

**Table 9:** Cross-dataset evaluation for AutoBot with and without CMTF. The Brier-minFDE metric is reported. The best results are in bold. Lower values are better.

Evaluation	Model	Training	
		nuScenes	Argoverse2
nuScenes	AutoBot	4.39	3.95
	AutoBot w/CMTF (λ = 0.5)	**3.90**	**3.86**
Argoverse2	AutoBot	2.37	1.75
	AutoBot w/CMTF (λ = 0.5)	**1.99**	**1.66**
WOMD	AutoBot	7.46	4.69
	AutoBot w/CMTF (λ = 0.5)	**7.29**	**4.55**

**Table 10:** Evaluation results on nuScenes and WOMD. The best results are in bold, and the second-best results are underlined. ↓ indicates that lower values are better.

Method	Variant	nuScenes				WOMD			
		brier_fde ↓	minADE6 ↓	minFDE6 ↓	miss_rate ↓	brier_fde ↓	minADE6 ↓	minFDE6 ↓	miss_rate ↓
MTR	−	4.009	1.495	3.361	0.496	11.438	4.334	10.659	0.825
MTR w/CMTF	λ = 0.3	3.931	1.458	3.286	**0.474**	11.617	4.201	10.838	0.821
MTR w/CMTF	λ = 0.5	3.926	1.456	3.277	0.492	**10.386**	**3.785**	**9.633**	**0.782**
MTR w/CMTF	λ = 0.6	**3.757**	**1.388**	**3.108**	0.479	12.810	4.908	12.028	0.814
AutoBot	−	3.945	1.588	3.255	0.488	4.689	1.554	3.990	0.576
AutoBot w/CMTF	λ = 0.1	3.795	**1.458**	**3.105**	0.479	4.733	1.550	4.033	0.571
AutoBot w/CMTF	λ = 0.3	3.820	1.510	3.129	0.474	4.840	1.601	4.140	0.578
AutoBot w/CMTF	λ = 0.5	**3.683**	1.528	3.168	**0.468**	**4.555**	**1.500**	**3.851**	**0.561**
AutoBot w/CMTF	λ = 0.6	4.094	1.578	3.400	0.495	4.734	1.532	4.048	0.569

## Data Availability

The data presented in this study are openly available in the public repositories of nuScenes, Argoverse2, and WOMD; the access links are provided in Section 4.1.1.
